# The impact of responsible gambling framing on people with lived experience of gambling harm

**DOI:** 10.3389/fsoc.2023.1074773

**Published:** 2023-03-07

**Authors:** Sarah Marko, Samantha L. Thomas, Hannah Pitt, Mike Daube

**Affiliations:** ^1^Institute for Health Transformation, Faculty of Health, Deakin University, Geelong, VIC, Australia; ^2^Faculty of Health Sciences, Curtin University, Perth, WA, Australia

**Keywords:** gambling, responsible gambling, affected others, public health, framing

## Abstract

**Background:**

The framing of health issues influences how people think about and respond to these topics. Gambling has largely been framed as an issue of personal responsibility, with the gambling industry, governments and some researchers promoting responsible gambling strategies as a way to address gambling harm. While there is evidence that the internalization of personal responsibility can negatively impact gamblers, this study aimed to explore how people who have experienced gambling harm interpret and apply personal responsibility frames and ‘gamble responsibly' messages in their lives.

**Methods:**

In-depth semi-structured interviews were conducted via Zoom and telephone with 15 gamblers who had been harmed by their own gambling and six affected others who had been harmed by someone else's gambling. This study was informed by public health and critical qualitative approaches to inquiry. The data were analyzed using reflexive thematic analysis.

**Results:**

Four themes were constructed from the data. First, gamblers and affected others generally conceptualized gambling and gambling harm as being the responsibility of the individual because it was perceived as the outcome of individual behavior. Second, they attempted to apply responsibility to their own experience either as gamblers who tried to stop or reduce their gambling, or affected others who felt responsible for helping the gambler in their lives. Third, gamblers and affected others were negatively impacted when it was perceived the gambler could not ‘control' their gambling or had not done enough to take responsibility. Finally, gamblers and affected others recommended responsible gambling strategies be reframed to be more effective at addressing gambling harm.

**Conclusion:**

This study provides evidence further supporting research demonstrating that personal responsibility frames may have unintended or negative consequences for gamblers and affected others. It underscores the need to reframe public messages about gambling away from responsible gambling, and toward research-based messages that can complement broader legislative changes and other measures to protect individuals.

## 1. Introduction

Message framing is recognized as having a powerful influence on how health and social issues are defined (Entman, 1993). Framing influences how different population groups perceive the causes and consequences of complex health and social issues, and can impact on the range of policy solutions that are used to respond (Koon et al., 2016). As people construct meaning and knowledge from information in the world around them, framing can be used strategically to influence how people and policy makers think about and respond to health and social issues (Burr, 2015; Molder et al., 2021). There has been a specific focus in the academic literature on how harmful commodity industries, such as the tobacco and alcohol industries, have used personal responsibility framing about the consumption of their products (and associated harms) in ways that serve to protect their interests and reputation (Friedman et al., 2015; Maani Hessari and Petticrew, 2018; Maani et al., 2022). For example, prevention programs developed by the tobacco industry promoted and improved the industry's image and interests rather than changing smoking behaviors (Henriksen et al., 2006). Similarly, campaigns developed by the alcohol industry have been found to be ambiguous and the meanings of the messages interpreted in multiple ways by community members, with some interpreting their messages as promoting alcohol consumption (Jones et al., 2017). The World Health Organization (2017) has stated that careful consideration is needed about how health issues are framed because messages must be clear, relevant and empowering in order to be effective.

More recently, researchers have criticized the strategies used by the gambling industry, governments, and some researchers to frame gambling as an issue that is largely associated with personal responsibility (van Schalkwyk et al., 2019). This framing and the associated responsible gambling paradigm positions gambling as a recreational activity that the majority of the adult population can choose to engage with in a responsible way without experiencing harm (Hancock and Smith, 2017a; Orford, 2019). This responsible gambling framing has been increasingly criticized by commentators from the public health community for creating the perception that gambling problems only occur if individuals misuse products and engage in irresponsible behavior (Hancock and Smith, 2017a; Miller and Thomas, 2018; Francis and Livingstone, 2021). Researchers have argued that this approach implies that there is a ‘right' way to gamble which is fun and controlled (the ‘responsible gambler'), and a wrong way to gamble which is uncontrolled and harmful (the ‘problem gambler' or ‘gambling addict') (van Schalkwyk et al., 2022:2). Responsible gambling also assumes that individuals make rational and informed decisions about how they use gambling products (Francis and Livingstone, 2021). Researchers have argued that such messages have limited impact because the focus on individuals as decision-makers overlooks the role of the gambling industry and governments in the creation of harmful gambling (Marko et al., 2022).

Research conducted in Australia (Marko et al., 2022), Canada (Savard et al., 2022) and Sweden (Samuelsson and Cisneros Örnberg, 2022) shows that gamblers largely view gambling as an issue of individual responsibility. This internalizing of personal responsibility among gamblers is a concern because it contributes to the problematization and stigmatization of people who experience problems with gambling (Alexius, 2017; Miller and Thomas, 2018). This is because it creates an “*overly simplistic”* narrative of the causes of harm that does not accurately reflect the factors that influence how people make decisions about their behavior (Hodgins, 2021:876-877). Furthermore, qualitative research has shown that people who experience problems with gambling do not perceive responsible gambling messages as being effective at changing an individual's behavior (Miller and Thomas, 2018).

Given the criticisms of responsible gambling framing, there has been a shift within the gambling industry and some government organizations toward safer gambling messages which promote strategies to keep gamblers safe while they gamble (Davies et al., 2022). As the focus remains on individuals, there is no evidence for any meaningful difference between these and responsible gambling messages. Research into the effectiveness of ‘responsible' or ‘safer' messages has largely focused on pop-up messages that aim to disrupt individual gambling behaviors. A recent systematic review found that while such messages may have some short-term impacts on behavior, there is limited evidence for sustained behavior change (Bjørseth et al., 2021). These messages may also be overwhelmed by the omnipresent commercial messages about gambling through advertising and marketing and does not address the harms experienced by others such as families and social networks (Victorian Auditor-General's Office, 2021). However, responsible or safer gambling frames still dominate the messages that are given about strategies to minimize gambling harm. This has been referred to by Alexius (2017:472) as “*direct consumer responsibilization.”* For example, recent industry harm minimization campaigns have encouraged gamblers to “*Take a sec before you bet”* (Sportsbet, 2021), “*Take time to think”* (Betting and Gaming Council, 2022), and “*Have a game plan”* (American Gaming Association, 2022). Similar messages which promote responsible gambling are communicated in public education campaigns that are run by governments even when these are framed as public health campaigns (Cassidy, 2020, p. 93). Examples of taglines from such campaigns include “*Embrace moderation”* (Alberta Gaming, Liquor and Cannabis, 2021), “*Stop gambling in time!”* (The Brussels Times, 2021), and “*You've got the power”* (Department of Justice Community Safety, 2015) [which bears a striking resemblance to the widely criticized “*I've got the power”* school-based education program about smoking sponsored by Philip Morris in the late 1990s (Chapman, 2001)]. Recent reports have criticized the impact and effectiveness of these messages in public health approaches to preventing gambling harm. For example, van Schalkwyk et al. (2021a) found that the gambling industry's claims about the effectiveness of their “*When the fun stops, stop”* campaign were not backed by evidence, and that the framing used in the campaign may create the perception that gambling harm is a problem for a minority of gamblers rather than a broader public health issue.

There are, however, four clear gaps in the current literature. The first is that there is limited research that explores how gamblers may attempt to apply information about responsible gambling to their own lives. Second, there is limited understanding of the alternative frames that gamblers think could be used in public messages about gambling. Third, while there is some evidence that affected others view gambling harm as the result of individual behavior (McCarthy et al., 2022c), no research to date has specifically explored how responsible gambling frames may impact on the perceptions held by people who have been directly impacted by someone else's gambling (‘affected others'). Finally, Alexius (2017) suggested that the members of a gambler's social network may reinforce that the gambler is responsible for harm if they have been taught that gambling is a personal responsibility. However, there has been limited research regarding how affected others view responsible gambling messages and how they apply these in their lives.

In order to address these gaps, this study drew on data collected as part of a broader qualitative study that sought to understand how people who have been harmed by gambling conceptualize gambling risk and harm. The aim of the current study was to explore how people who have experienced gambling harm interpret and apply personal responsibility frames and ‘gamble responsibly' messages in their lives. The study was guided by four research questions:

1. How do those affected by gambling harm broadly conceptualize responsibility for gambling harm?
2. How do they apply personal responsibility strategies to their own or someone else's gambling?
3. What impact does personal responsibility have on those who experience gambling harm?
4. What alternative frames do they think could be used in public messaging about gambling?

## 2. Methods

### 2.1. Approach to inquiry

This study used both public health and critical qualitative approaches to inquiry. A public health approach recognizes that a range of individual, socio-cultural, environmental, commercial and political factors contribute to gambling harm, and that this harm can impact individuals, their families, and communities (Goyder et al., 2020). This approach recognizes that gambling harm disproportionately impacts vulnerable communities, and that the gambling industry exploits these vulnerabilities (Rae and Fell, 2022). In order for solutions to be equitable, Rae and Fell (2022) argue that strategies cannot focus on clinical or individual interventions but on the broader drivers of harm. A critical qualitative approach to inquiry was also used as it aligns with the public health approach and seeks to advance social justice by studying power and inequality (Charmaz, 2017). Going beyond interpretation, critical researchers identify opportunities to advocate for change to address inequalities, inequities, and injustices, and focus on the powerful institutions, public policies, and discourses that contribute to these issues (Denzin, 2017). These approaches were appropriate given the unequal power wielded by the gambling industry and governments in framing gambling and influencing strategies to address harm, as compared to those impacted by gambling harm (Hancock and Smith, 2017b; van Schalkwyk et al., 2021b).

Experts by Experience (EbyE) have been recognized as valuable stakeholders who have unique perspectives due to their lived experience of gambling harm, and who should be included in considerations about gambling research, education and treatment (Nyemcsok et al., 2021). The authors formed an EbyE Advisory Group of four individuals who were either former gamblers or affected others, and who provided feedback on the project. This included ensuring the interview was respectful and reflected the language used by people with lived experience of harm. Members of the EbyE Advisory Group were compensated for their time with a $50 grocery voucher.

### 2.2. Recruitment and data collection

The study aimed to recruit 15-20 adult participants, which was similar to previous critical qualitative inquiries in relation to gambling (McCarthy et al., 2021; Nyemcsok et al., 2022b). To be included in the broader study, participants needed to be adults who lived in Australia and had experienced housing-related harm as a result of their own current or past gambling (‘gamblers') or someone else's gambling (‘affected others'). Gamblers were included as they are the target audience for responsible gambling programs, and affected others were included because to our knowledge there had been limited research into their perspectives despite their being impacted by gambling harm (Goodwin et al., 2017).

A range of convenience, purposive and snowball sampling methods were used to recruit participants. The members of the EbyE Advisory Group and other people with lived experience of gambling harm who were known to the researchers shared the study advertisement flier on social media sites, and with their personal and professional networks, including peer support and advocacy groups. Snowball techniques were also used as those who participated in the study were asked to share the study flier with their social network. Using multiple recruitment strategies provided diversity among gamblers in terms of how recently they had gambled. In order to recruit a greater number of affected others, the researchers also sought permission from service providers, including mental health counselors and financial counselors, to distribute information about the study to their clients who may have been eligible to participate. There was a particular focus on ensuring that women were well represented in our recruitment strategies. While men generally have higher rates of ‘problem gambling,' the literature shows that women experience more financial stress from gambling (Koomson et al., 2022). However, researchers have highlighted that they are often excluded or underrepresented in gambling studies (McCarthy et al., 2019). It was also important to ensure that women's experiences were represented in the study as both gamblers and affected others (McCarthy et al., 2022b,c). All participants provided informed consent and were offered a $50 grocery voucher. Ethical approval was obtained from the Deakin University Human Ethics Research Committee (2021-003).

Semi-structured, audio-recorded interviews lasting ~60 min were conducted via Zoom or telephone by Authors One and Two. The interview guide used with gamblers focused on several themes: their history with gambling, views about the risks and harms associated with gambling, experiences of gambling related harm, and strategies to prevent gambling harm. The interviews with affected others focused on the same topics, however they were asked to reflect on their understanding of the gambling history of someone in their life, and their own experiences of gambling related harm. Throughout the data collection process, the interviews became more focused as further questions were asked in relation to key topics and ideas from discussions with previous participants (Hennink et al., 2020). Data collection ended when there was enough information from the interviews to answer the research questions (Malterud et al., 2016).

### 2.3. Data analysis

Automated transcripts were generated by the Zoom software for online interviews, and audio recordings were transcribed by Author One for the telephone interviews. All transcripts were checked and edited for accuracy. The six phases of Braun and Clarke's (2021) reflexive thematic analysis were used to interpret and construct themes from the data. This process of interpretation occurred during and after the data collection process. Notes were taken following each interview about the key topics which were discussed, and data familiarization continued throughout the transcription process. The coding and theme development focused on the research questions. The data were coded based on the semantic (surface level) and latent (deeper more nuanced) meanings. These codes were grouped to identify patterns across the interviews and the research questions were used to construct preliminary themes. Rather than making comparisons between gamblers and affected others, the aim of the analysis was to present themes that reflected a central unifying concept that represented the different perspectives of the participants both within and across groups (Braun and Clarke, 2022). This allowed for a focus on diverse experiences as well as any similarities between different participants. This approach was important because we did not start with an assumption that all gamblers and all affected others would have similar experiences. Rather we aimed to construct a more nuanced interpretation of the lived experiences of participants. The researchers met regularly to discuss the interpretation of the data, construction of the themes, and the implications of the findings.

## 3. Results

A total of twenty-one people participated in this study, including fifteen gamblers and six affected others. The mean age was 48.05 years (SD = 15.19). Approximately half of the participants were male (*n* = 11), and all of the affected others were women. Just over half of the participants lived in Victoria (*n* = 12) and the majority identified as Australian. Table 1 provides an overview of the demographics for each participant. The participants had a range of experiences with gambling and gambling harm. Some gamblers had not gambled for many years, while others had ceased gambling more recently or currently gambled at the time of the interview. Most affected others' family members currently gambled. Several participants including gamblers and affected others discussed how they or their family member had contemplated or attempted suicide as a direct result of their experience of gambling harm. This included two affected others who had a sibling die by gambling-related suicide.

**Table 1:** Participant demographics.

**Gamblers**				
**Age**	**Gender**	**State**	**Ethnicity**	**Main gambling products used**
25	Male	VIC	White Jewish/Australian	Casino table games, sports, horse, and dog race betting
31	Male	QLD	Australian	Casino table games, poker machines, sports, and horserace betting
37	Male	WA	New Zealander	Casino table games, sports betting
40	Male	VIC	Australian Chinese	Horserace betting, casino table games
40	Male	VIC	Australian	Sports and horserace betting
43	Male	NSW	Australian	Sports and horserace betting
49	Male	VIC	Australian	Sports betting
55	Male	QLD	Australian	Horserace betting
56	Male	VIC	Portuguese	Horserace betting, poker machines, lottery
57	Male	VIC	Australian	Horserace betting
62	Male	NSW	Indigenous Australian	Poker machines
64	Female	VIC	Australian	Poker machines
69	Female	VIC	Australian	Poker machines
71	Female	VIC	Australian	Poker machines
73	Female	VIC	English/Anglo-Saxon	Poker machines
**Affected others**				
**Age**	**Gender**	**State**	**Ethnicity**	**Person whose gambling they were harmed by**
25	Female	NSW	Albanian	Mother
29	Female	ACT	Australian	Husband
30	Female	WA	Australian	Brother
48	Female	VIC	Australian	Ex-husband
49	Female	WA	Australian	Son
56	Female	VIC	Australian	Sister and brother

Table 2 provides a summary of the four themes that were constructed from the data and demonstrates, where appropriate, any key similarities and differences between the perspectives and experiences of gamblers and affected others.

**Table 2:** Summary of the themes according to responses by gamblers and affected others.

**Theme**	**Gamblers**	**Affected others**
Theme one: Conceptualizing the role of responsibility in gambling harm	• Many gamblers viewed gambling harm as a personal responsibility. • Harm was viewed as the result of their own gambling behavior. • Many did not think about the risks associated with gambling. • Some perceived gambling was a recreational activity that most people engaged in without experiencing harm. • Some suggested addiction impacted gamblers' ability to make rational decisions. • The gambling industry was also viewed as being responsible for gambling harm.	• Some affected others viewed gambling harm as a personal responsibility. • Harm was viewed by some as the result of gamblers being irresponsible. • Some perceived gambling was a recreational activity that most people engaged in without experiencing harm. • Some suggested addiction impacted gamblers' ability to make rational decisions. • The gambling industry was also viewed as being responsible for gambling harm.
Theme two: Applying responsible gambling to their own or someone else's gambling	• Gamblers tried to take responsibility when they felt they could no longer control their gambling. • Tools to set gambling limits or self-exclude from gambling accounts and venues were easy to bypass. • Profession support services were hard to access and were not necessarily helpful.	• Affected others felt they were responsible for helping their family member when their gambling became harmful. • Some tried help the gambler understand the impact of their gambling and take control of their gambling. • Some took financial responsibility to limit the impact gambling addiction had for the gambler, including bearing these outcomes themselves.
Theme three: Impact and shame of not meeting the expectations of responsible gambling	• Many blamed themselves for experiencing gambling harm and felt ashamed for not being able to control their gambling. • Some kept their gambling secret due to fear of judgment.	• All affected others had been lied to by their family member about their gambling. • There was tension between affected others and their family member with some feeling frustrated or helpless.
Theme four: Reframing and moving beyond responsible gambling messages	• Gamblers criticized messages about the need to take personal responsibility because it overlooked their attempts to change their gambling behavior. • Personal responsibility messages were perceived as being ineffective at preventing harm and were not taken seriously or seen was being relevant. • These messages were seen as being hypocritical when coming from the industry that provides and promotes gambling products. • Gamblers suggested that messages should provide realistic portrayals of harms associated with gambling. • Some perceived that the government should implement practical action to reduce harm in addition to messaging.	• Personal responsibility messages were perceived as being ineffective at preventing harm. • These messages were seen as being hypocritical when coming from the industry that provides and promotes gambling products. • Affected others suggested that messages should provide realistic portrayals of harms associated with gambling. • Some perceived that the government should implement practical action to reduce harm in addition to messaging.

### 3.1. Theme one: Conceptualizing the role of responsibility in gambling harm

Participants (including both gamblers and affected others) generally conceptualized gambling and gambling harm as being the responsibility of the individual. This was because gambling harm was perceived as primarily the outcome of individual behavior. Some affected others perceived that their family members were responsible for the harm experienced because they gambled money they could not afford to lose, or they had not taken responsibility to change their gambling behaviors. Similarly, some gamblers conceptualized their own experiences of harm as the consequence of their own behavior because they did not budget or limit the money they gambled, and instead continued to gamble when they perceived they should have stopped. A few gamblers suggested that their harmful gambling was influenced by their personal belief that they could control the outcome of the bet or they could “*beat the system”*:

…probably nothing would have stopped me from doing what I did because I had this irrational belief that I could beat the system, that I'd be the one to make a living out of this, become rich and my family would benefit, my wife would benefit. -49-year-old male gambler

Some participants viewed gambling as being an inherently risky activity and perceived that each individual needed to assess the risk associated with their own gambling: “*…if you're not prepared to take a risk, don't do it”*. However, many gamblers acknowledged that they did not initially think about the risks their own gambling posed to their own wellbeing, or how to manage these risks when they first began gambling. Many gamblers framed gambling as a dichotomy; either it was an activity that was recreational and low risk for most people, or it was harmful and addictive for others. This division between recreational and harmful gambling also contributed to the perception that “*it's not dangerous for everybody,”* and that most people could gamble recreationally because they were able to maintain control. Some gamblers described low-risk recreational gambling as being able to gamble occasionally, gamble for social or leisure reasons, and “*walk away from it”* when needed. For example, the following participant perceived that some people could enjoy gambling and stop when they need to:

I think there are people out there who just have a bit of social fun and can do it and can enjoy it and can stop when they need to stop... I've seen people, you know, that can do it and can have a bit of enjoyment out of it and make a social event out of it and that type of thing. -57-year-old male gambler

Similarly, some affected others also spoke about gambling as being an activity that was only harmful to some people. Some of these participants reflected on their own experiences with gambling or seeing others in their social network gamble without engaging in harmful behavior. For example, the following affected other did not perceive that her current partner was at risk of developing a gambling addiction because he engaged in it recreationally and with small amounts of money:

I have a new partner now. He gambles but he'll gamble like a dollar and he's very careful with his money. So, it's something that he does recreationally but, um, just for enjoyment really, just to talk about it to mates… I have no concern that he's going to have an addiction problem at any point in the future. -48-year-old female impacted by her ex-husband's gambling

Some participants (including both gamblers and affected others) perceived that individuals who experienced gambling addiction and the subsequent harm were different to those who could gamble recreationally. It was suggested that some people were more likely to engage in this type of gambling due to a range of individual factors that influenced their behavior and choices. For example, this included believing that they could win or control the outcome of the bet, having an “*addictive personality,”* or being “*predisposed”* to addiction due to genetics or the experiences of family members. The following participant compared himself to people he saw in the venues he attended who he perceived were able to gamble without experiencing harm and suggested that he was different from them because he will “*never do that”*:

…the older guys and that's there once a week. You know, they have two or three beers and they'll be betting one or two dollars every race or whatever. They look at the form guide. Now that's their entertainment and they're able to do that and that's fine. They're not losing a lot or maybe they lose every single week but that's in the budget, you know. I can't, I can't, I can never do that, you know. That's just me. I can never ever do that. -55-year-old male gambler

While gambling harm was framed as the consequence of individual behavior, there was also the perception among some gamblers and affected others that individuals were unable to make rational decisions about their gambling once addiction had occurred. Gamblers explained that it was as though “*I'd been taken over,”* and “*your mind is being controlled.”* This contributed to the perception among some that individuals could not be held responsible for their behavior which resulted from addiction. For example, the following participant rejected the idea of telling people experiencing addiction to take personal responsibility because it was not possible:

And people say, “Oh, you shouldn't have to legislate. It's all about personal responsibility”. But once you've slipped down that slide of addiction, accountability really, it's too late. You know, you can't say—you know, my family's initial reaction was, “Just don't go.” Ha, and I've still got a brother-in-law who talks that way but it's not that easy. -71-year-old female gambler

In addition to the individual's role in gambling harm, some gamblers and affected others commented that the gambling industry was also responsible for gambling harm due to the provision and promotion of products that caused addiction and harm. This included criticisms regarding the extent to which gambling was marketed. Gambling marketing was perceived as creating “*temptation,”* particularly for gamblers who wanted to stop gambling. For example, a few gamblers and affected others suggested that the gambling industry used inducements sent via email and push notifications to mobile phones to encourage people to gamble. There was also acknowledgment among some that gambling products, particularly poker machines, were designed to maximize losses, including that the machines were programmed “…*to slaughter, not bloody just skim a little bit* [of money] *off the top,”* and prevented people from thinking about the risks associated with their gambling:

It's a lovely feeling, you know. Music in the background, dings and dah dah dah. And you know, it's a very, very pleasant environment to feel hypnotized in. And I think that's—You know, it's only when I went out of the place that it would hit me when I walked out. -73-year-old female gambler

### 3.2. Theme two: Applying responsible gambling to their own or someone else's gambling

Most gamblers described how they had attempted to take responsibility for their gambling, including trying to stop or reduce their gambling. This was typically done when they felt they could no longer control their gambling, or it was creating harm. Many gamblers discussed how they attempted to apply personal responsibility strategies but described unexpected challenges. First, while tools were available to support behavior change, gamblers explained ways in which these were ineffective. Tools to set limits or self-exclude were easy to work around and they could continue gambling. As these tools for online gambling products typically applied to individual companies, gamblers could simply open accounts with other companies. It was also suggested that the self-exclusion register at physical venues was rarely enforced by staff and, when it was, they could go to another venue. One participant recalled confronting venue staff when they did not enforce her self-exclusion and how she was told it was her responsibility not to attend the venue:

I renewed (the self-exclusion) and it was only two or three weeks, and I went into another venue one or twice, three times. Nothing was said to me. And when I said to them, “I put this in place, why aren't they doing it?” they said, “Well, it's still your responsibility not to go in there.” So, the responsibility is always on us as the gambler to do it. -69-year-old female gambler

Second, some gamblers had sought help from professional services such as mental health practitioners, financial counselors, general practitioners, and Gambler's Help services. However, these were not easy to access and, even when accessed, were not necessarily helpful. It was suggested that professionals did not always understand gambling harm or addiction: “*I've spoke*[n] *to counselors who don't understand”*. Others noted that they experienced delays in accessing professional support because services were underfunded, difficult to access, or did not offer the assistance they needed. One participant explained that his difficulties in accessing help had contributed to his ongoing gambling issues and led him to “*be more irresponsible”* with his gambling:

So, the amount of times I try to get help, to be responsible, just led me to be more irresponsible because I just couldn't get it. So, I just felt stuck… I have more chance of winning than I do getting help. -40-year-old male gambler

There was a perception among affected others that they had a responsibility to help the gambler in their life when their gambling had become harmful. When gamblers were unable or unwilling to reduce their gambling, affected others felt as though they had to intervene and help the gambler take responsibility. While a few affected others had confronted their family member in an appeal to get them to change their behavior, others explained that their family member had approached them and asked for support to reduce their gambling. A few affected others reflected that their motivation to support their family member was the “*love”* they had for them, and the participants wanted to ease the harm their family member experienced. For example, one participant prioritized supporting her brother by loaning him “*a lot of money”* because she perceived that this prevented him from seeking alternative and riskier sources of money:

But I didn't even hesitate, like bang, you know, “Here you go. As much as you need, just tell me”. Because I wanted him to like have a safe money place, you know, not like do something stupid or get involved with drugs or something… I was like $10,000 is a lot of money, but I thought in the context of (my brother) feeling safe, it's nothing. -30-year-old female impacted by her brother's gambling

A few affected others also suggested that there were external pressures to take responsibility for intervening in their family member's gambling. One participant explained how as a child she was told by the adults in her life that she needed to “*convince* [her] *mother”* that her gambling was harmful and needed to change, while another participant felt pressured to help her ex-husband while they were married because the broader community would judge her for his actions:

…that's the thing, when you're married to someone like that, they represent you and then people start to think that you're like that when, you know, you're just trying to survive. At some point you kind of think “Well, you're married to them, you try to get them well”. -48-year-old female impacted by her ex-husband's gambling

One way some affected others perceived that they were taking responsibility was by trying to help the gambler take personal responsibility for their gambling. This included helping them to understand the impact their gambling had on themselves and those around them, and helping the gambler to self-exclude from gambling products or access professional support. A few affected others had taken responsibility for their family member's finances in order to reduce their access to money and prevent gambling or to ensure their broader financial responsibilities were met:

He had a young family and I had to go in and take over. I was only in my twenties. And so, it was a long time ago. I had to take over his finances for about three months. Just, you know, I paid their bills. I paid everything. And that's money that's still owed to me. -56-year-old female impacted by her siblings' gambling

Taking on financial responsibility meant some affected others also bore responsibility for the broader financial outcomes relating to the gambling addiction. Some affected others went to extreme efforts to limit the financial outcomes of the gambling addiction. For example, the mother of a gambler contacted a bank that loaned her son money that she believed he would not be able to pay back, while many affected others had contributed their own money in order to help the gambler meet their other financial responsibilities or repay debts. This often impacted their own financial situation when these funds were not repaid. One participant also highlighted how she continued to be negatively impacted by her ex-husband's gambling addiction because she had taken financial responsibility during their marriage and was now unable to take out loans or a mortgage:

I may as well have laid all those bets because that's how life treats me. If I want a loan, whatever, I might as well have had the addiction myself. I don't think that's fair… Why do I then have to suffer for the rest of my life not being able to buy again or get a loan because all I've done is give up all that I had to try and help someone who does have an addiction? Well, where's my reward? There is none. -48-year-old female impacted by her ex-husband's gambling

### 3.3. Theme three: Impact and shame of not meeting the expectations of responsible gambling

Many gamblers spoke about the impact of not being able to successfully implement responsibility strategies to control their gambling. These experiences were viewed by some gamblers as a personal fault and they used moralizing and judgmental language to describe themselves and others who experienced addiction—“*absolute losers,”* and “*liars and cheaters”*. A few gamblers commented that they could not explain or justify their gambling behavior because it did not reflect how they behaved in other areas of their lives or how they saw themselves. This contributed to feeling as though their experience of gambling harm meant there was something wrong with them that they could not explain: “*you just can't understand why you did it because you're not that stupid generally.”* There was shame and embarrassment associated with the inability to control their gambling and experiencing harm. The emotional impact of this was reflected by a few gamblers who stated that they had contemplated suicide, while others described instances where they became angry or cried in response to their gambling. While one participant described the “*utter despair and frustration”* she felt, the following participant explained how he “*hated”* how he lied and stole due to his gambling but that it had become a “*compulsion”*:

I'd lost again and I just went to the toilet and burst out crying. And I was like, “Why am I crying? What's going on here? What's wrong here?” I was so disconnected from myself. Like I hated being a gambling liar and a thief. I really hated it, but it was habitual. It was an obsession and a compulsion. -25-year-old male gambler

Some gamblers explained how they hid their gambling from their social networks and at times socially isolated themselves because they found it difficult to be honest about their gambling. There was a fear of being open about their experiences of harm due to the perception that they would be judged for not being able to control their gambling. For a few gamblers, the fear of judgment was confirmed as they were told to “*just stop”* by people they had disclosed their gambling to, which reinforced to them to keep their experiences a secret. One participant explained how the shame associated with his gambling led him to become deceptive and contributed to further harm:

And I lied and was deceptive and just couldn't tell the truth and couldn't be me and couldn't, you know, tell people how I was feeling. You know, my feelings and emotions were always hidden. And I just couldn't be me. It just felt like it was all an act. Like I was an actor being another person, um, and I wasn't being me. And I was conscious of it, but I was too afraid to actually say anything because, you know, the shame behind it. And that's how the pile and pile got larger and larger and larger over time. -40-year-old male gambler

Affected others suggested that they were negatively impacted when the gambler in their lives hid or lied about their gambling. All of the affected others stated they had been lied to, and most acknowledged that gamblers might not be honest about their gambling or be able to ask for help. However, many also acknowledged that this was most likely due to the associated shame. This created tension between the participants and their family member who gambled. Some felt frustrated when they were lied to or suggested that the gambler had not tried hard enough to change their gambling behaviors. Others indicated a helplessness when they could not support the gambler because they were not being honest. There was also a perception that they needed to be careful when talking to their family member because saying the wrong thing could contribute to their shame, trigger them to gamble more, or make them angry:

…we were all on tenterhooks always. So, if (my son's) sort of hidden away with his phone and whatnot, we're all whispering out here saying “Do you think he's gambling?” and then I say “Okay, I'm going to go and ask him” and just by asking it sets off the whole range of emotions where he probably was looking at something and so because of the guilt, he then gets angry that we haven't trusted, that we can't trust him. -49-year-old female impacted by her son's gambling

### 3.4. Theme four: Reframing and moving beyond responsible gambling messages

Despite their own attempts to apply personal responsibility strategies, gamblers and affected others were largely critical of messages based on the responsible gambling and personal responsibility framing. Critical phrases were used to describe these messages including “*a load of rubbish,” “bullshit,”* and “*pathetic”* because they were perceived as being ineffective at preventing harm. A few gamblers commented that it was not possible to ‘gamble responsibly' either because gambling was “*irresponsible,”* or because they were not able make rational decisions while gambling. Some gamblers also suggested that messages such as ‘gamble responsibly' were meaningless and were not taken seriously by people who gamble. For example, the following participant suggested that ‘gamble responsibly' messages were treated as a joke among people that gamble:

It's left it open for back-handed comments or like people that joke with their mates. Like really, if you look at the online forums and stuff about responsible gambling comes up, it's a fucking joke. -40-year-old male gambler

Participants often suggested that it was hypocritical for the gambling industry to tell people to take personal responsibility while providing and promoting addictive products. It was suggested that responsible gambling messages were a “*token gesture”* and were used by the gambling industry to deflect responsibility and appear as though they were responding to gambling harm. The following participant perceived that the gambling industry did not actually want people to change their gambling behavior:

…it's just to cover up because like “We're more than happy to feed it to you day in, day out, whenever we can.” It's just like oh no—unfortunately I don't know the words to describe it. It's like we'll create this program um just saying, “Okay, we want you to gamble but we're going to pretend that we don't”. -31-year-old male gambler

Many participants (including gamblers and affected others) suggested that there was a need to reframe messages away from responsible gambling toward something that would be more effective. The most common recommendation was the provision of honest and realistic information about the harms associated with gambling products via public messaging, and education in schools to prevent future harm. There was a perception that this would enable people experiencing harm to speak up and get help by destigmatizing harm and addiction, while also encouraging people to rethink their gambling and discourage young people from trying it in the first instance. Some also suggested that the messaging should be similar to that from other public health areas such as tobacco, alcohol and road accidents. They perceived that these “*hard-hitting,” “off-putting,”* and “*brutal”* campaigns were effective and recommended that a similar approach should be used in relation to gambling. For example, the following affected other suggested that people understood the harms relating to cigarettes because of public messaging, and that messaging about gambling should portray realistic harms that people can relate to their life rather than “*superficial”* messages:

(People know) if you smoke, you can get cancer. But I don't know that people really can appreciate—like you might sort of see an ad saying “don't gamble” but you don't really see someone—like the smoking ads, it might be, I don't know, a big hole in their throat or whatever. But you don't really see people talking about “I've lost everything. I've lost my home. I've lost my family.” There's not really that kind (of message), it doesn't go to that level. It's more a superficial level of what the warning signs are but there's not really a personable example provided for people. -48-year-old female impacted by her ex-husband's gambling

Some gamblers and affected others perceived that strategies beyond public messaging were needed in order to prevent harm and suggested practical actions that the government could implement. These included reducing the accessibility of gambling products, banning or reducing gambling marketing, reforming the self-exclusion register to make it more robust, and introducing mandatory deposit limits. Participants did not typically suggest that gambling should be prohibited and instead focused on creating safe gambling environments to protect those who gamble from the risks of harm. There was also a particular focus on the need to protect people who were vulnerable to or experience gambling addiction:

Now I'm very much aware in my own mind that every human being deserves protection, especially someone who's lost their ability to protect themselves which is an addict. They need more protection than anybody. -73-year-old female gambler

## 4. Discussion

**Figure 1:**
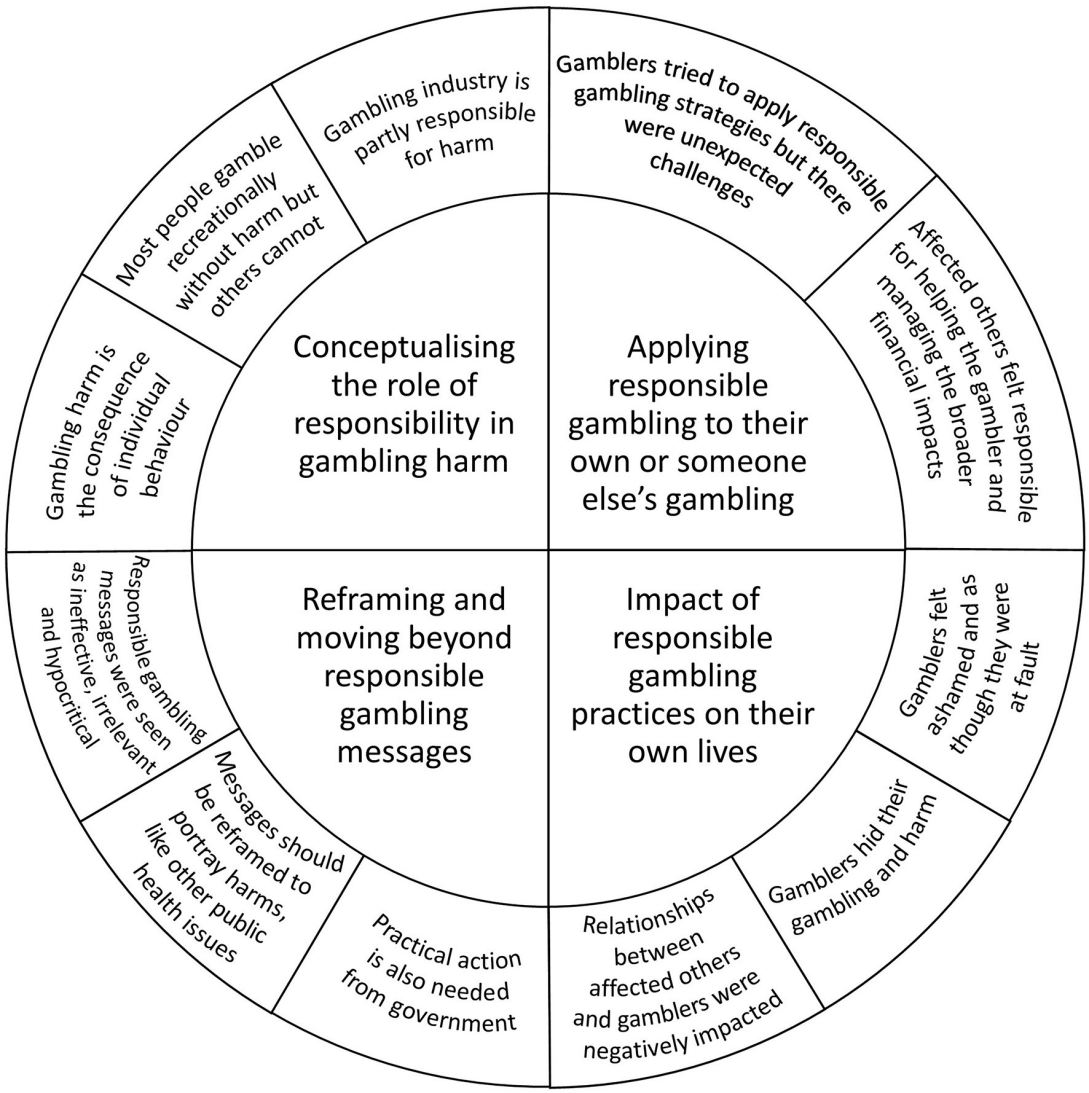
Understanding how gamblers and affected others conceptualize and apply personal responsibility and the impact this has in their lives.

This study aimed to explore how people who have experienced gambling harm interpret and apply personal responsibility frames and ‘gamble responsibly' messages in their lives, and the alternative frames they think could be used in public messaging. Figure 1 depicts a model of the themes that were constructed from the data and descriptions of the key findings from each theme. The four themes relate to how gamblers and affected others conceptualized the role of responsibility in gambling harm, how they applied responsible gambling to their own or someone else's gambling, the impact of not meeting the expectation of responsible gambling, and their perceptions about reframing and moving beyond responsible gambling messages.

The findings of this study raise three points of discussion relevant to the research questions.

First, this study demonstrated that gamblers generally conceptualized gambling as an issue of personal responsibility despite some acknowledgment of the gambling industry's role in contributing to harm. This supports the findings of previous studies indicating that gamblers have internalized personal responsibility (Marko et al., 2022; Samuelsson and Cisneros Örnberg, 2022; Savard et al., 2022). This study also shows that the focus on personal responsibility for action still remains after experiencing harm and that even when the role of the gambling industry is acknowledged, blame is ultimately placed on the individual. This study also provides the new perspective that personal responsibility has been internalized by affected others with some perceiving that their family member was responsible for the harm that occurred. This shows that the dominant personal responsibility framing impacts not only gamblers but those around them and highlights the importance of including affected others in discussions about responsibility for harm. As public health researchers have clearly recognized that a broad range of socio-cultural, environmental, commercial and political determinants contribute to gambling harm (Goyder et al., 2020), the findings from this study appear to show that efforts to frame gambling as being about the individual, rather than the gambling industry or public policy, have been effective. As people construct meaning based on the information around them (Burr, 2015), framing can influence how people conceptualize issues by emphasizing specific information (Entman, 1993). This study provides further evidence for researchers' concerns about the impact of the dominance of personal responsibility framing and lack of counter-framing in and around the gambling market (Alexius, 2017; Marko et al., 2022).

Second, this study provides evidence that supports calls to reframe messages and discourses relating to gambling away from personal responsibility and “responsible gambling” frames (Francis and Livingstone, 2021; Maani et al., 2022). Researchers have previously suggested that these frames create the perception that there is a responsible/controlled and irresponsible/uncontrolled way to gamble (Marko et al., 2022; van Schalkwyk et al., 2022). This study adds to the existing literature by demonstrating that people who perceive they engage in irresponsible or uncontrolled gambling try to correct this by acting upon the messages that have been given about personal responsibility. It further shows that affected others also try to act upon these messages by helping the gambler to change their behavior, or take responsibility to limit the financial impact of the gambling. This demonstrates that affected others have unique experiences that are distinct from those of gamblers and reinforces the need to include them in discussions about message framing which involve the lived experience community.

This study also shows that gamblers' and affected others' efforts to apply responsibility appear to be largely disempowering because they create negative feelings, stigma and tension between gamblers and affected others. This is particularly evident when there is the perception that the gambler has not been successful in taking responsibility. While this has been seen in other areas of public health such as tobacco and food where people blame themselves for their poor health outcomes (Hamann et al., 2014; Pearl and Lebowitz, 2014), there remains a need for further research regarding the impact this has on affected others in the gambling area. This study also demonstrates that gamblers and affected others recommend reframing messages about portrayals of gambling harm. While messages about harms have been used in regard to tobacco, these framed the products as the cause of harm to highlight why individuals should not smoke, and also provided messages of encouragement to acknowledge the difficulties when trying to stop (Bayly et al., 2019). By comparison, the Australian Government recently announced new taglines to replace “gamble responsibly” messages; however the new taglines continue to focus on individual decision making such as asking gamblers to consider what “*gambling* [is] *really costing you”* (Visentin, 2022). The recommendations from participants in this study suggest these messages do not go far enough in reframing the message away from blaming gamblers and may contribute to the disempowerment of people who experience harm. Reframing gambling harm as the consequence of a problematic and harmful industry rather than individual behaviors may reduce the shame and stigma associated with experiencing gambling harm. As with tobacco (Durkin et al., 2022), independent research is urgently needed to identity which messages will be most effective as part of a comprehensive public health approach, and how these messages should be disseminated to the public. Previous tobacco and alcohol research has highlighted concerns about industry-funded campaigns (Henriksen et al., 2006; Jones et al., 2017); this study also indicates that messages that are perceived as coming from the gambling industry are not taken seriously by gamblers. Consideration is needed regarding the most effective sources of information and methods for communicating harm prevention messages to the public.

Finally, this study showed that in addition to reframing messages about gambling, people who have experienced gambling harm clearly support a range of strategies which are consistent with the public health approach to harm prevention. This aligns with previous evidence that gamblers also want governments to take legislative steps to restrict the gambling industry (Marko et al., 2022; McCarthy et al., 2022a; Nyemcsok et al., 2022a), as they have done alongside reframing messaging in other public health areas such as tobacco (Chapman and Freeman, 2008). In the Australian state of Victoria where many of the participants for this study resided, the Public Health and Wellbeing Act recognizes that governments have a “*a significant role in promoting and protecting the public health and wellbeing…*[and] *promoting conditions in which persons can be healthy”* (Parliament of Victoria, 2008:s4). For government action to be comprehensive, it must use multiple evidence-based population-level strategies to target the different factors that contribute to gambling harm.

There were two limitations associated with this study. First, the number of affected others included in the study were relatively low despite attempts to recruit a greater number. Second, we were unable to recruit affected others who were men which may unintentionally reinforce gendered stereotypes regarding women as affected others. Addressing these limitations in future studies to involve a more diverse range of affected others would provide a greater understanding of the similarities and differences in experiences between and within gamblers and affected others. This would provide a more nuanced understanding of how responsible gambling frames impact different groups' experiences of harm. Consideration is needed regarding how best to recruit affected others. However, the difficulties related to participant recruitment for this study may relate to the narrow focus of the broader research project which specifically sought to recruit people who had experienced housing-related harm as a result of gambling.

## 5. Conclusion

This study demonstrates how those who have a lived experience of gambling harm internalize responsibility for gambling harm and attempt to apply responsibility to their own experiences. The framing of gambling as an issue of personal responsibility has dominated public messaging from the gambling industry, governments and some researchers. This study provides evidence for the negative impact these frames have on gamblers and affected others and underscores the need to reframe messages about harm. Public communication about gambling should move away from messages about individualized ‘responsible gambling' strategies to preventing harm, and toward evidence-based messages that will complement broader legislative and other changes to protect individuals and the community.

## Data availability statement

The datasets presented in this article are not readily available because participants explicitly consented to only have their data shared with the immediate research team.

## Ethics statement

The studies involving human participants were reviewed and approved by Deakin University Human Ethics Research Committee (2021-003). The patients/participants provided their written, verbal, and informed consent to participate in this study.

## Author contributions

SM and ST conceptualized and designed the study and conducted data collection. SM conducted the main qualitative analysis and produced the first draft of the manuscript. All authors contributed significantly to manuscript revision, read, and approved the final version.
